# Competition-level and positional differences in match running performance of Vietnamese professional soccer players: a GPS-based season analysis

**DOI:** 10.3389/fspor.2026.1890629

**Published:** 2026-07-24

**Authors:** Viet Nam Huynh, Tuan Hung Pham, Rangsarit Jamrern, Piyathida Thongchai, Phornpot Chainok

**Affiliations:** 1 Danang Sport University, Danang, Vietnam; 2 Faculty of Physical Education, The University of Danang, Danang, Vietnam; 3 Faculty of Sport Science, Burapha University, Chonburi, Thailand

**Keywords:** athlete monitoring, composite performance score, external load, high-speed running, running performance

## Abstract

**Introduction:**

This study aimed to quantify the match-running performance of Vietnamese professional football players utilizing global positioning system (GPS) technology and investigate variations based on competition level and position and establish a composite performance score (CPS) framework for player assessment and categorization.

**Methods:**

A longitudinal observational study was executed throughout the 2024-2025 season, encompassing 57 official matches involving six professional clubs from V-League 1 and V-League 2, comprised 497 player-match observations from 160 outfield players. External-load variables were gathered utilizing 10 Hz GPS devices and encompassed total distance (TD), maximum velocity (Vmax), high-speed running distance (HSR), sprint distance and accelerations/decelerations. Statistical analyses included independent-samples t-tests, one-way ANOVA with Tukey HSD post hoc comparisons and correlation analyses. V-League 1 players displayed significantly greater HSR, sprint distance, Vmax, accelerations and decelerations (*p* < 0.05) than V-League 2 players but TD was similar between competition levels.

**Results:**

There were significant differences between positions for TD, TD/90, Vmax, HSR and sprint distance (all *p* < 0.001) with forwards showing the greatest high-intensity locomotor outputs and defenders covering the greatest running volumes. The performance of Vietnamese players in HSR and sprint-related areas was significantly lower than elite European benchmarks. CPS classification indicated that the majority of observations were classified as Average (57.5%) with Elite classifications only accounting for 3.1% of observations.

**Background:**

These findings indicate that high-intensity locomotor capacity is an important physical determinant in professional Vietnamese football and that the CPS framework provides a practical multidimensional tool for performance profiling, individualized training and longitudinal athlete monitoring.

## Introduction

Modern football is characterized by increasingly complex physical, physiological, and tactical requirements, requiring players to consistently execute high-intensity movements interspersed with short recovery intervals during competitions. Contemporary match analyses reveal that elite outfield players generally cover between 10 and 13 km during a 90-min match, while performing significant amounts of high-speed running (HSR), sprinting, accelerations, decelerations, and swift directional changes (1–3). Moreover, recent studies from major European leagues and international competitions have shown consistent rises in match intensity, especially in high-speed running and sprinting activities, indicative of the changing tactical and transitional dynamics of contemporary football (4–6).

The ability of high-intensity running has become an important consideration in competitive performance, as critical offensive and defensive actions frequently occur during short, quick movements (7, 8). The incorporation of wearable microtechnology and global positioning system (GPS) devices has significantly improved researchers' and practitioners' capacity to measure external load in realistic competitive environments (9, 10). In contrast to conventional time-motion analysis, modern 10 Hz GPS systems provide enhanced reliability and sensitivity in quantifying running performance metrics, such as total distance (TD), maximum velocity (Vmax), high-speed running (HSR), sprint distance and acceleration/deceleration profiles (11–13).

The application of GPS technology has greatly enhanced the comprehension of location variances in the needs of football matches. Current evidence consistently indicates that locomotor demands differ based on tactical roles and positional duties during competition (14, 15). Midfielders normally achieve the largest total distance (TD) due to their constant engagement in both offensive and defensive transitions, while forwards usually exhibit the greatest volumes of sprinting and high-speed running (HSR) linked to breakthrough attacking maneuvers (16). Defenders typically demonstrate lower total running distances but may engage in numerous high-intensity accelerations during pushing and recovery activities (17).

Besides positional variation, competition level substantially impacts physical performance characteristics. Players in higher-standard levels consistently exhibit increased HSR exposure, sprint frequency, and match intensity compared to those in lower divisions (18). Longitudinal analyses indicate elite competitions have shown a consistent rise in high-speed activities over the past decade, possibly due to tactical development, enhanced physical preparation, and increased transitional tempo (2, 3). Although such findings have been widely documented in European football, evidence from Asian professional leagues is relatively scarce, despite significant cultural, environmental and developmental difference that may affect physical match performance (19).

Many of the recent studies in Asia attempted to characterize the match-running demands of professional football players by the utilization of GPS technology. Studies from the Japanese J League and Chinese Super League have indicated diminished TD and HSR values compared to elite European standards, implying possible differences between regions in tactical approach, training practices and competitive intensity (20–23). Notwithstanding the rapid development of football throughout Southeast Asia, empirical data elucidating the physical exigencies of Vietnamese professional football is limited. Accordingly, the establishment of normative GPS-derived performance profiles for Vietnamese professional football players is a significant practical and scientific priority.

Recent advances in sports analytics and athlete monitoring have highlighted the significance of composite and data-driven processes that can synthesize several performance markers into a single interpretable index (24). The performance in modern football is influenced by the interplay of aerobic workload, sprinting ability, repeated high-intensity efforts and the mechanical requirements linked to accelerations and decelerations (25). As a consequence, composite performance score (CPS) frameworks have arisen as a potentially advantageous method for integrating several external-load factors, such as total distance (TD), high-speed running (HSR), sprint distance, accelerations, decelerations and maximal velocity, into a standardized multidimensional index. These frameworks may offer coaches and sports scientists a more thorough assessment of player performance, enabling longitudinal monitoring, positional benchmarking and evidence-based training prescriptions (9).

The CPS models are often constructed by a series of sequential processes that include variable selection, score standardization, and physiological weighting methods. Variables are selected based on their relevance to football-specific locomotor criteria, while standardization techniques like z-scores or T-scores facilitate the integration of variables recorded in disparate units (16). Weighting procedures are subsequently implemented to represent the relative physiological significance of each metric, with high-intensity activities such as sprinting and high-speed running typically receiving greater emphasis due to their strong correlation with crucial match actions and competitive success (7, 8). Notwithstanding the increasing interest in multidimensional monitoring systems, CPS frameworks are still inadequately developed in Southeast Asian football, especially in Vietnam, where GPS-based match analytics remain constrained. Furthermore, inconsistencies in speed thresholds, weighting structures and classification criteria continuously restrict comparisons between research and competitive levels (12).

Consequently, the development of a standardized CPS framework for Vietnamese professional football might significantly enhance athlete monitoring and performance profiling by creating context-specific criteria for domestic and international competition. The present study aimed to quantify the match physical performance of Vietnamese professional football players using GPS technology, examine differences according to competitive level and playing position and develop a CPS framework for player classification and longitudinal monitoring.

## Materials and methods

### Participants

A total of 57 official matches from the 2024–2025 competitive season were analyzed, comprising 160 professional male football players and 497 individual player-match observations from six clubs competing in V League 1 and V League 2. The dataset included four clubs from V League 1 (303 observations) and two clubs from V League 2 (194 observations). Only observations from outfield players completing at least 45 min of match participation were retained for analysis, consistent with established procedures in observational football match-analysis research (14, 15). Goalkeepers were excluded because their locomotor and tactical demands differ substantially from those of outfield players. Matches presenting substantial GPS signal interruptions (>5 min) or poor-quality data were excluded from the final dataset.

Furthermore, only GPS files meeting the manufacturer's acceptable quality-control threshold and observations with plausible maximum velocity (Vmax) values following unit normalization (2.8–12.5 m·s^−1^) were included in the analysis. External-load data were collected using 10 Hz Catapult Vector and STATSports Apex Pro systems, both of which have demonstrated acceptable validity and reliability for football-specific locomotor monitoring (12, 13). Playing positions were assigned according to each player's primary tactical role during competition, as determined by team formation and consistent on-field behavior throughout match play. Match observation and tactical analysis were used to verify positional classification and ensure consistency with positional responsibilities (14).

Outfield players were classified as Outfield players from V-League 1 and V-League 2 were categorized according to their primary playing position as defenders (DEF; V-League 1: *n* = 34, V-League 2: *n* = 56), midfielders (MID; V-League 1: *n* = 54, V-League 2: *n* = 76), and forwards (FWD; V-League 1: *n* = 74, V-League 2: *n* = 96). Competitive level was categorized according to participation in either V League 1 or V League 2. Based on the Participant Classification Framework proposed by Aaron K. McKay et al. (26), the present sample may be classified as Tier 4 athletes because all participants competed within the highest professional football divisions in Vietnam.

### Procedures

All participants were informed about the objectives, methodologies, risks and benefits related to the study before to their involvement, and written informed consent obtained from all participants. This study was conducted in compliance with the Declaration of Helsinki and received approval from the institutional research ethics committee. Match-running performance was assessed throughout 57 official matches over the 2024–2025 competitive season featuring clubs in V League 1 and V League 2. All matches were conducted in accordance with official competition regulations on standard football pitches, with typical environmental changes associated with professional football in Vietnam.

External-load data were collected only throughout match play; warm-up periods, half-time intervals, bench time, and post-match activities were excluded from the analysis. Subjects were observed utilizing 10 Hz GPS devices, specifically the Catapult Vector S7 (Catapult Sports, Melbourne, Australia) and STATSports Apex Pro (STATSports Group, Newry, Northern Ireland), both of which incorporate tri-axial accelerometers, gyroscopes, and magnetometers, and have previously exhibited satisfactory validity and reliability for football-specific locomotor monitoring (12, 13).

Post-match GPS data were extracted and exported utilizing Catapult OpenField and STATSports Apex software for further processing and analysis, in accordance with accepted protocols in applied football performance monitoring (9, 13). The following external-load variables were identified including total distance (TD), total distance normalized to 90 min (TD/90), maximum velocity (Vmax), high-speed running (HSR; 5.5–7.0 m·s^−1^), HSR as a percentage of total distance (HSR%), sprint (≥7.0 m·s^−1^), accelerations (≥3 m·s^−2^), decelerations (≤ −3 m·s^−2^) and an explosive Index which defined as the sum of accelerations and decelerations relative to total distance covered (km).

The selected variables together represent essential indications of football-specific locomotor, neuromuscular and mechanical demands during match play (1, 17, 25). Speed zones and locomotor thresholds were established based on criteria typically utilized in elite football GPS studies and were consistently applied throughout all matches to maintain methodological consistency (11, 14, 19). High-speed running and sprinting activities were explicitly incorporated according to their significant correlation with decisive offensive and defensive maneuvers, serving as essential markers of competitive football performance (7, 8).

**Figure 1:**
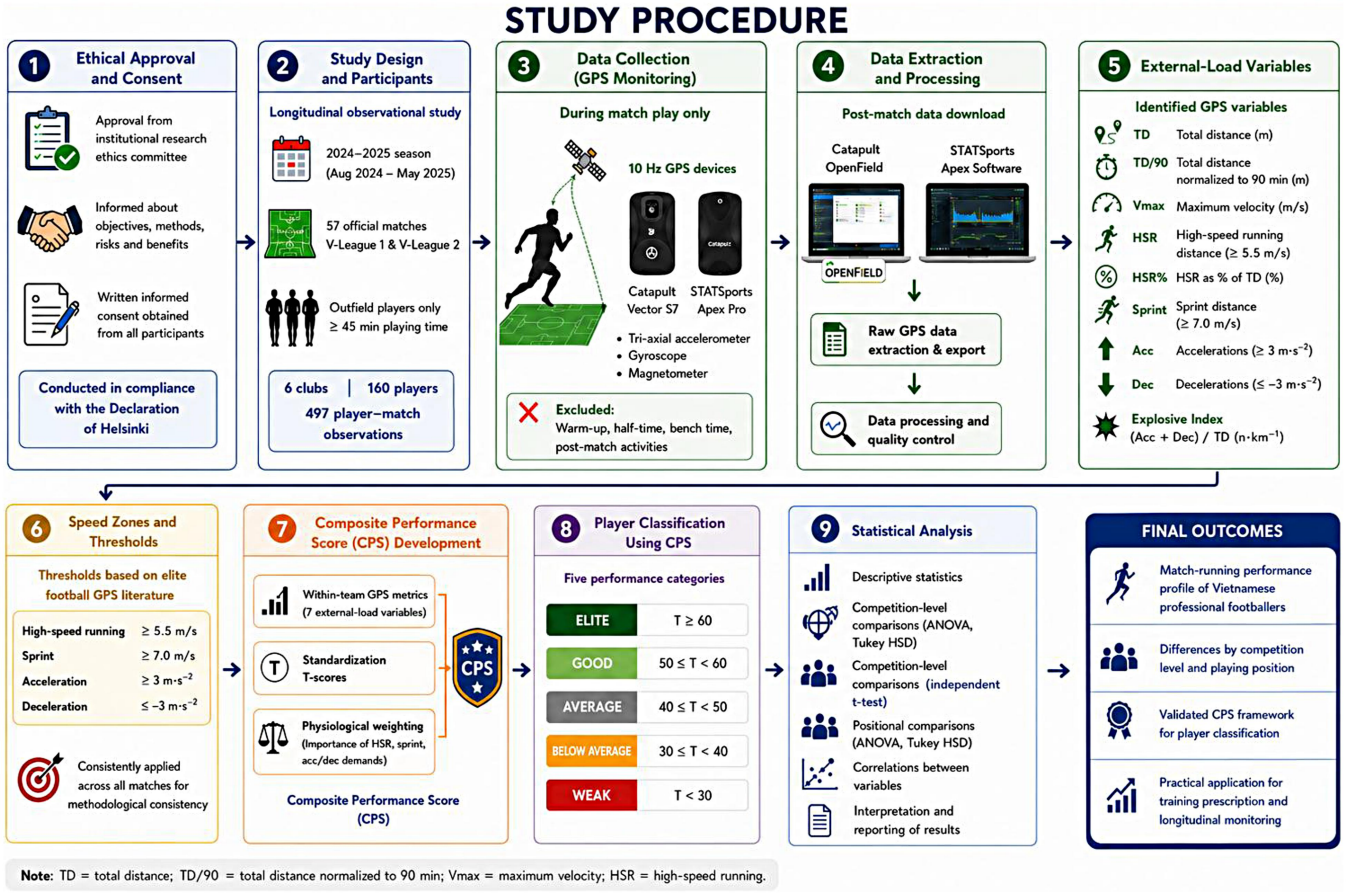
Schematic overview of the study procedures including GPS monitoring, external-load analysis, CPS development, player classification and statistical analyses in Vietnamese professional football.

A composite performance score (CPS) framework was subsequently developed using standardized T-scores derived from within-team GPS metrics and physiologically weighted according to their relevance to football-specific locomotor performance. The CPS model integrated seven external-load variables and classified player performance into five categories: Elie, Good, Average, Below average and Weak. The weighting structure was informed by previous literature emphasizing the importance of high-intensity running, sprinting capacity and acceleration–deceleration demands in elite football performance (15, 25, 27) (Figure 1).

### Statistical analysis

Descriptive statistics are presented as mean ± standard deviation (SD), median (Mdn), interquartile range (P25–P75), and coefficient of variation (CV). Normality was assessed using the Kolmogorov–Smirnov test, and homogeneity of variances was evaluated using Levene's test. Differences in GPS-derived match-running variables between competition levels (V-League 1 and V-League 2) were examined using independent-samples t-tests. Effect sizes were calculated using Cohen's d and interpreted as trivial (<0.20), small (0.20–0.49), moderate (0.50–0.79) and large (≥0.80) (28). Positional differences in GPS-derived external-load variables were examined using one-way analysis of variance (ANOVA) with Tukey HSD *post hoc* comparisons when significant main effects were identified. Effect sizes for ANOVA were quantified using partial eta squared (*η*^2^p) and interpreted as small (0.01), medium (0.06) and large (0.14) (29).

Associations among GPS-derived performance variables were assessed using Pearson product–moment or Spearman rank correlation coefficients, depending on data distribution. Correlation magnitudes were interpreted as trivial (0.00–0.25), weak (0.26–0.49), moderate (0.50–0.69), strong (0.70–0.89) and very strong (0.90–1.00) (30). The Composite Performance Score (CPS) was generated using standardized within-team T-scores combined with physiologically weighted GPS metrics and used to classify player performance into five categories: Elite, Good, Average, Below Average and Weak. All statistical analyses were performed using SPSS version 29 (SPSS Inc. Company, Chicago, IL, USA) Statistical significance was accepted at *p* < 0.05.

## Results

Descriptive statistics for the complete dataset (*n* = 497) are presented in Table 1. After normalization to 90 min, mean total distance covered during a match was 7,553.10 ± 2,485.50 m (TD/90 = 8,274.50 ± 2,522.10 m). Mean maximum velocity was 7.80 ± 0.81 m·s^−1^, mean HSR distance was 350.90 ± 211.00 m (4.65 ± 2.69% of TD), and mean sprint distance was 79.00 ± 63.00 m. The highest coefficients of variation were observed for sprint distance (79.80%) and HSR (60.10%), reflecting the large between-player variability characteristic of high-intensity match activities.

**Table 1:** Descriptive statistics of GPS-derived match-running variables in Vietnamese professional soccer players (complete dataset).

Variable	Mean ± SD	Median	P25–P75	Min–Max	CV (%)
TD (m)	7,553.10 ± 2,485.50	7,444.0	5,866.90–9,282.80	90.00–15,256.30	32.90
TD/90 (m)	8,274.50 ± 2,522.10	8,180.10	6,660.30–10,054.40	250.00–16,341.20	30.50
Vmax (m·s^−1^)	7.80 ± 0.81	7.78	7.25–8.28	5.22–10.02	10.30
HSR (m)	350.90 ± 211.00	323.90	184.30–468.40	4.00–1,339.10	60.10
HSR (%)	4.65 ± 2.69	4.17	2.57–6.31	0.00–16.61	58.00
Sprint (m)	79.00 ± 63.00	63.20	33.10–110.70	0.00–382.10	79.80
Accelerations (*n*)	87.00 ± 43.00	83.00	61.00–112.00	10.00–271.00	45.20
Decelerations (*n*)	95.00 ± 46.00	75.00	55.00–102.00	14.00–267.00	45.60

Values reflect the complete dataset (*n* = 497).

TD, Total distance; TD/90, TD normalized to 90 min; Vmax, Maximum velocity; HSR, High-speed running (≥5.5 m·s^−1^); HSR%, HSR as a percentage of TD; CV, Coefficient of variation.

Total distance covered was not different between divisions (mean difference = 16.40 m, *p* = 0.34, trivial effect). On the other hand, V-League 1 players exhibited higher HSR than V-League 2 players (mean difference = 206.90 m, *p* < 0.001, d = 1.10). Maximum velocity was also higher in V-League 1 (7.92 ± 0.89 vs. 7.61 ± 0.61 m·s^−1^, *p* = 0.003, d = 0.40). Similarly, sprint distance, accelerations and decelerations were all greater in V-League 1 with moderate effect sizes (d = 0.74–0.77). On the other hand, the TD/90 was higher in V-League 2 (9,072.70 ± 2,271.00 vs. 7,758.60 ± 2,561.00 m, *p* = 0.01, d = −0.53) (Table 2).

**Table 2:** Competition-level differences in GPS-derived match running variables.

Variables	V-League 1 (*n* = 303)	V-League 2 (*n* = 194)	Mean diff	95% CI	*p*-value	Cohen's *d*
TD (m)	7,556.20 ± 2,553.00	7,539.80 ± 2,412.00	16.40	(−17.30, 50.10)	0.34	0.01 (Trivial)
TD/90 (m)	7,758.60 ± 2,561.00	9,072.70 ± 2,271.00	−1,314.10	(−2,292.80, −335.40)	0.01	−0.53 (Moderate)
Vmax (m·s^−1^)	7.92 ± 0.89	7.61 ± 0.61	0.31	(0.11, 0.51)	0.00	0.40 (Small)
HSR (m)	431.70 ± 208.60	224.80 ± 141.90	206.90	(92.80, 321.00)	<.001	1.10 (Large)
Sprint (m)	96.80 ± 69.40	51.30 ± 37.60	45.50	(24.20, 66.80)	<.001	0.77 (Moderate)
Accelerations (*n*)	98.10 ± 45.60	69.60 ± 30.40	28.50	(15.20, 41.80)	0.01	0.74 (Moderate)
Decelerations (*n*)	106.80 ± 47.90	75.40 ± 33.80	31.40	(17.60, 45.20)	0.01	0.76 (Moderate)

Descriptive cells are observation-level mean ± SD. Cohen's *d* magnitude: trivial <0.2, small 0.2–0.5, moderate 0.5–0.8, large > 0.8.

The TD, TD/90, Vmax, HSR distance and sprint distance had significant positional differences (all *p* < 0.001) with moderate to large effect sizes (*η*^2^p = 0.12–0.27). Defensive players presented the highest TD (8,213.00 ± 1,938.00 m) and TD/90 (8,723.00 ± 1,402.00 m), whereas forwards displayed the highest Vmax (8.06 ± 0.72 m·s^−1^), HSR distance (404.00 ± 220.00 m) and sprint distance (112.60 ± 79.00 m). The midfielders displayed intermediate values for most of the running variables. In contrast, no significant positional differences were observed for accelerations (*p* = 0.67, *η*^2^p = 0.01) and decelerations (*p* = 0.98, *η*^2^p = 0.01), suggesting similar short-duration locomotor actions across playing positions (Table 3).

**Table 3:** Positional differences in GPS-derived match-running variables among outfield players.

Variable	DEF	MID	FWD	*F*	*p*-value	*η*^2^p
TD (m)	8,213.00 ± 1,938.00	8,063.00 ± 2,078.00	7,916.00 ± 2,152.00	41.54	<.001	0.27
TD/90 (m)	8,723.00 ± 1,402.00	8,691.00 ± 1,788.00	8,615.00 ± 2,044.00	32.18	<.001	0.22
Vmax (m·s^−1^)	8.03 ± 0.56	7.89 ± 0.67	8.06 ± 0.72	36.78	<.001	0.25
HSR (m)	306.00 ± 184.00	331.00 ± 186.00	404.00 ± 220.00	20.90	<.001	0.16
Sprint (m)	55.60 ± 48.80	73.20 ± 57.90	112.60 ± 79.00	33.92	<.001	0.12
Accelerations (*n*)	81.00 ± 42.00	79.00 ± 41.00	87.00 ± 43.00	0.40	0.67	0.01
Decelerations (*n*)	88.00 ± 45.00	89.00 ± 46.00	88.00 ± 44.00	0.02	0.98	0.01

DEF, defenders; MID, midfielders; FWD, forwards; *η*^2^p partial eta squared (small = 0.01, medium = 0.06, large = 0.14).

**Figure 2:**
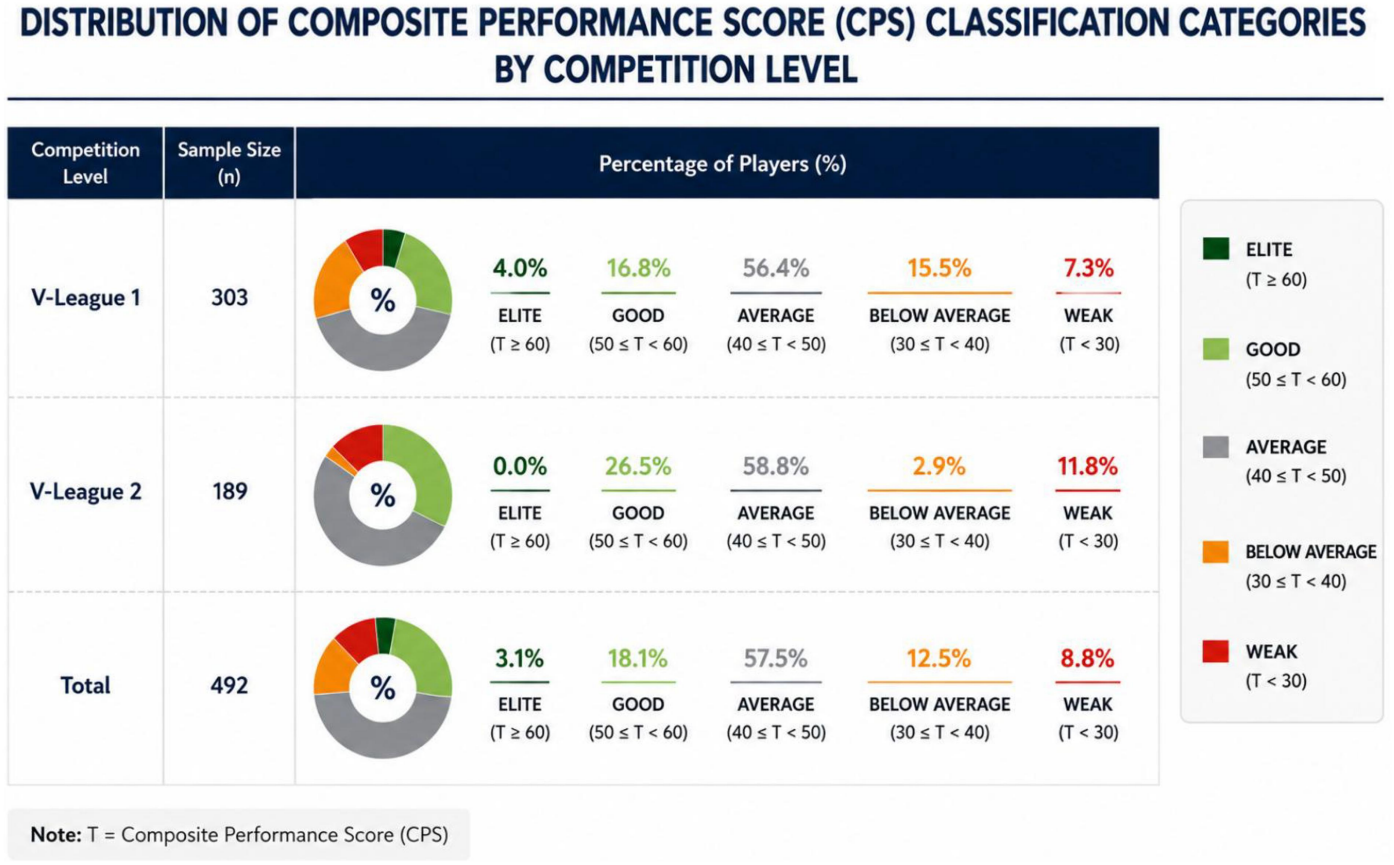
Distribution of composite performance score (CPS) classifications across competition levels in Vietnamese professional football players.

For the CPS, the competition levels were distributed differently across the CPS classification categories. Most player-match observations in V-League 1 were classified as Average (56.4%), and in V-League 2 it was 58.8%. V-League 1 had a higher percentage of Elite (4.0% vs. 0.0%) and Below Average classifications (15.5% vs. 2.9%), while V-League 2 had a higher percentage of Good (26.5% vs. 16.8%) and Weak performances (11.8% vs. 7.3%). Overall, 57.5% of observations were classified as Average, followed by Good (18.1%) and Below Average (12.5%), while Elite performances comprised only 3.1% of all observations (Table 4 and Figure 2).

**Table 4:** Distribution of composite performance score (CPS) classification categories by competition level.

Competition level	Elite	Good	Average	Below average	Weak
V-League 1	4.00%	16.80%	56.40%	15.50%	7.30%
V-League 2	0.00%	26.50%	58.80%	2.90%	11.80%
Total	3.10%	18.10%	57.50%	12.50%	8.80%

CPS, Composite performance score derived from within-team standardised T-scores. Values are the percentage of player-match observations within each classification category.

## Discussion

The present study observations on one of the first season-long GPS-based analyses of match-running performance in Vietnamese professional football and provides novel insights into competition-level and positional locomotor demands in a Southeast Asian context. Overall, Vietnamese professional players had lower high-intensity running and sprint metrics compared to elite European reference values, despite relatively similar total running volumes. Moreover, the level of competition was mainly differentiated by high intensity locomotor variables such as HSR, sprint distance and maximum velocity, while total distance was similar between V-League 1 and V-League 2. Positional analyses also confirmed that forwards performed the greatest high speed and sprint activities, while defenders accumulated higher total running volumes. In general, these results indicate that high-intensity locomotor capacity may be an important physical discriminant of competitive performance in Vietnamese professional football.

One interesting finding is the lower HSR and sprint values of Vietnamese professional players compared to elite European levels which is may be linked to differences in tactical intensity, physical preparation and pace of competition between leagues. Earlier studies in the English Premier League and UEFA competitions have shown that elite players have a tendency to cover greater amounts of sprinting and repeated high-intensity movements during match play (2, 3, 14, 15). Longitudinal analyses of elite European football have also shown progressive increases in HSR and sprint activities over recent decades, likely associated with faster transitions and increased tactical pressing demands (4, 5). The present study shows that the Vietnamese players achieved around 27%–36% of the HSR values generally reported in elite European competitions, but with relatively similar TD values. These findings partially concur with previous studies in Asian leagues including the Chinese Super League which also observed reduced high intensity locomotor outputs compared to European football (20–23). Accordingly, the current findings may suggest that the ability to run at high intensity is still a key developmental objective in Vietnamese professional football.

Moreover, the present findings showed that competition level was mainly a differentiator for high-intensity locomotor variables, rather than overall running volume. TD was similar between V-League 1 and V-League 2. However, V-League 1 players showed significantly higher HSR, sprinting distance, maximum velocity and acceleration–deceleration activity. These findings are in agreement with previous studies demonstrating that higher-level competitions are associated with higher exposure to high-intensity actions and transitional demands (15, 18). Similarly, Barnes et al. (2) and Bush et al. (3) showed that modern elite football is increasingly dependent on repeated explosive locomotor actions rather than absolute running volume. High-intensity actions are often related to decisive offensive and defensive scenarios, such as counterattacks, pressing actions and goal-scoring opportunities (6–8). Consequently, the ability to repeat high-intensity efforts may be an important physical characteristic that differentiates higher-level Vietnamese professional players.

Interestingly, total running distance was generally comparable to values reported in some higher-level competitions, whereas high-speed running and sprint performance was substantially lower (2, 3, 15). This pattern suggests that the key difference may not be the total volume of locomotor activity but the ability to perform and sustain high-intensity actions during match play. Thus, the physical gap between Vietnamese professional football and elite international standards may be more related to high-intensity locomotor capacity than total running output (15). Similarly, the higher TD/90 in V-League 2 should be interpreted with caution. A greater running volume does not necessarily imply a greater match intensity and may reflect differences in tactical organization, ball possession patterns, match tempo or game structure between levels of competition (18, 22, 23).

For the positional analyses perspective, the results of present study are generally in line with previous GPS-based studies in elite football, which showed that forwards perform more explosive and sprint-related actions linked to attacking penetrations and transition phases (14–16). On the other hand, defenders and midfielders tend to have higher total running volumes, as they are frequently involved in the tactical organization and defensive coverage during match play (1, 3, 18). Besides, there were no significant differences in accelerations and decelerations between playing positions. This finding may suggest that current Vietnamese professional football has similar mechanical demands for short duration between tactical positions, possibly due to close tactical formations or frequent pressing tasks. In addition, Harper et al. (25) pointed out that accelerative and decelerative actions are important neuromuscular stressors in elite football and may not always present traditional positional patterns.

An additional novel aspect of the present study was the development of a CPS framework integrating multiple GPS-derived external-load variables into a multidimensional player classification system. Most of the observations were classified in the “Average category” while a small fraction was classified with the Elite classification. These findings may reflect the homogeneity of physical profiles within Vietnamese professional football and further support the limited exposure to elite-level high-intensity locomotor demands. Recent advances in athlete monitoring have highlighted the need for multidimensional performance models that can incorporate running volume, sprinting exposure and acceleration-deceleration demands into practical decision-making frameworks (9, 24, 25). The CPS approach implemented in the present study may represent a practical tool for longitudinal athlete monitoring, return to play evaluation and individual conditioning prescription for coaches and sports scientists. Moreover, from an applied perspective, the CPS framework may help coaches and sports scientists to facilitate more transparent benchmarking between teams, competition levels and positional groups, thereby enabling practitioners to identify individual strengths and locomotor deficiencies with greater precision.

These findings should be interpreted with caution due to the observational design of the study, which does not allow definitive causal inferences regarding the reported competition-level and positional differences. First, the data was obtained from only six professional clubs and may not represent the general physical characteristics of Vietnamese professional football. Therefore, the findings may not fully represent all Vietnamese professional football clubs. Second, as analyses were conducted at the player-match observation level, a degree of dependence among observations may have been present and should be considered when interpreting the findings. Third, the use of two different GPS systems, although both previously validated, may also have introduced small inter-device variability despite the standardized data-processing procedures.

Additionally, contextual match-related factors such as tactical formation, quality of opposition, match status, environmental conditions and fixture congestion were not controlled and may have influenced locomotor performance outcomes. Future studies should incorporate these contextual variables within multilevel or mixed-effects modelling frameworks to better understand their contribution to match-running performance. Finally, the CPS model was constructed only based on external-load measures obtained from GPS, without considering internal-load, physiological, technical or tactical indicators, which could compromise the multidimensional representation of football performance. Besides, the weighting structure was not derived from data-driven approaches, but from previous literature. Further studies should consider broader performance measures and also consider objective weighting methods such as the principal component analysis to further develop the CPS framework.

## Conclusion

The Vietnamese professional football players exhibited a markedly reduced high-intensity locomotor performance in comparison to elite international standards, especially in high-speed running and sprint-based activities, even though their total running volumes were approximately similar. Competition was primarily characterized by high-intensity external load factors, with positional analyses showing an increased demand for sprints and high-speed running in forwards and elevated running volumes in defenders. The findings underline the importance of high-intensity locomotor capacity as a key physical component of competitive football performance. The CPS framework developed in this study provides an innovative multidimensional approach to integrating GPS-derived external-load data within an efficient player-monitoring and classification system. Overall, these findings may be useful for the development of evidence-based training prescription, positional benchmarking and longitudinal athlete monitoring in the context of Vietnamese professional football, while providing also practical reference values for performance optimization and individualization of conditioning strategies.

## Data Availability

The original contributions presented in the study are included in the article/Supplementary Material, further inquiries can be directed to the corresponding author.
